# The Effect of *CYP, GST,* and *SULT* Polymorphisms and Their Interaction with Smoking on the Risk of Hepatocellular Carcinoma

**DOI:** 10.1155/2015/179867

**Published:** 2015-01-14

**Authors:** Stefania Boccia, Luca Miele, Nikola Panic, Federica Turati, Dario Arzani, Consuelo Cefalo, Rosarita Amore, Milutin Bulajic, Maurizio Pompili, Gianlodovico Rapaccini, Antonio Gasbarrini, Carlo La Vecchia, Antonio Grieco

**Affiliations:** ^1^Institute of Public Health, Section of Hygiene, Department of Public Health, Università Cattolica del Sacro Cuore, Largo Francesco Vito 1, 00168 Rome, Italy; ^2^IRCCS San Raffaele Pisana, Via della Pisana 235, 00163 Rome, Italy; ^3^Institute of Internal Medicine, Gemelli Hospital, Università Cattolica del Sacro Cuore, Largo Francesco Vito 1, 00168 Rome, Italy; ^4^Internal Medicine and Gastroenterology Unit, Complesso Integrato Columbus, Via Giuseppe Moscati 31-33, 00168 Rome, Italy; ^5^University Clinical-Hospital Center “Dr Dragisa Misovic-Dedinje”, Milana Tepica 1, 11000 Belgrade, Serbia; ^6^Department of Epidemiology, IRCCS Istituto di Ricerche Farmacologiche “Mario Negri”, Via La Masa 19, 20156 Milan, Italy; ^7^Faculty of Medicine, University of Belgrade, Dr Subotica 8, 11000 Belgrade, Serbia; ^8^Internal Medicine and Gastroenterology Division, Gemelli Hospital, Università Cattolica del Sacro Cuore, Largo Francesco Vito 1, 00168 Rome, Italy; ^9^Department of Clinical Sciences and Community Health, Università degli Studi di Milano, Via Festa del Perdono 7, 20122 Milan, Italy

## Abstract

*Aim*. The aim of our study was to assess whether selected single nucleotide polymorphisms of *CYP1A1* and *2E1*, *GSTM1*, *GSTT1*, and *SULT1A1* influence susceptibility towards HCC, considering their interaction with cigarette smoking. *Methods*. We recruited HCC cases and controls among patients admitted to the hospital “Agostino Gemelli,” from January 2005 until July 2010. Odds ratios (OR) of HCC were derived from unconditional multiple logistic regression. Gene-gene and gene-smoking interaction were quantified by computing the attributable proportion (AP) due to biological interaction. *Results*. The presence of any *CYP2E1* ^*^ *5B* variant allele (OR: 0.23; 95% CI: 0.06-0.71) and *CYP2E1* ^*^ *6* variant allele (OR: 0.08; 95% CI: 0.01–0.33) was inversely related to HCC. There was a borderline increased risk among carriers of combined *CYP1A1* ^*^ *2A* and *SULT1A1* variant alleles (OR: 1.67; 95% CI: 0.97–3.24). A significant biological interaction was observed between *GSTT1* and smoking (AP = 0.48; 95% CI: 0.001–0.815), with an OR of 3.13 (95% CI: 1.69–5.82), and borderline significant interaction was observed for *SULT1A1* and smoking (AP = 0.36; 95% CI: −0.021–0.747), with an OR of 3.05 (95% CI: 1.73–5.40). *Conclusion*. *CYP2E1* ^*^ *5B* and *CYP2E1* ^*^ *6* polymorphisms have a favourable effect on the development of HCC, while polymorphisms of *GSTT1* and *SULT1A1* might play role in increasing the susceptibility among smokers.

## 1. Introduction

Hepatocellular carcinoma (HCC) is currently the sixth most common cancer and the third cause of cancer deaths worldwide [1]. Its prognosis remains poor, with a 5-year survival rate less than 20% in Europe [2]. Risk factors for HCC include infection with hepatitis B (HBV) and hepatitis C viruses (HCV), history of diabetes mellitus, nonalcoholic fatty liver disease and cirrhosis, heavy alcohol consumption, and cigarette smoking [3–5]. Coffee consumption appears to have a favorable effect [6]. Further, genetic factors appear to modulate the individual susceptibility as the siblings of HCC individuals are more prone to develop the HCC even in the absence of HBV infection [7]. So far, several single nucleotide polymorphisms (SNPs) have been investigated in association with HCC, with contradictory results [8].

As the liver is the main metabolic organ, the SNPs related to genes encoding carcinogen-metabolizing enzymes represent key target candidates for association analyses. Cytochrome P-450 (*CYP*) is a superfamily of monooxygenases responsible for phase I enzyme reactions, preparing substrates for phase II conjugation reactions, but they can also lead to the metabolic activation of toxic or carcinogenic compounds [9]. The glutathione S-transferases (*GSTs*) are a gene superfamily coding phase II enzymes that detoxify free radicals in tobacco smoke, products of oxidative stress, and polycyclic aromatic hydrocarbons [10]. Sulfotransferase (*SULT*) catalyzes sulfonate conjugation to detoxify the procarcinogens to metabolites, which are easily eliminated from the body [11]. Polymorphisms in these genes, their combination, and interaction with environmental factors have the potential to lead to increased susceptibility to HCC. While some studies investigated the effect of* GST* and* CYP* genes on HCC, as well as their combination and interaction with smoking [12, 13], little information is available on the effect of* SULT1A1*.

The aim of our study was to assess whether the selected SNPs of* CYP1A1* and* 2E1*,* GSTM1, GSTT1, *and* SULT1A1* genes influence individual susceptibility to HCC, also considering their combination and interaction with cigarette smoking.

## 2. Methods

### 2.1. Study Population

Study participants were recruited among patients admitted to the teaching hospital “Agostino Gemelli” of the Università Cattolica del Sacro Cuore (Rome, Italy) from January 2005 until July 2010, and eligibility was restricted to white individuals born in Italy. Cases were 221 patients with HCC recruited among subjects referred to the Outpatient Liver Unit of the hospital. The diagnosis of HCC was performed according the AASLD guidelines [14]. The control group included 290 patients from the same hospital with a broad range of diagnoses, enrolled during the same time period. In closer detail, around 50% of the controls were outpatients, and the remaining were patients undergoing surgical interventions (laparoscopic cholecystectomy, appendicitis, and inguinal hernia) or admitted for a wide spectrum of other nonneoplastic conditions (elderly patients on physical-therapy rehabilitation after stroke or orthopedic injuries). Written informed consent was obtained from all study subjects. The study was conducted according to the Declaration of Helsinki and was approved by the Ethical Committee of Università Cattolica del Sacro Cuore.

HCC cases and controls were interviewed by trained physicians using a structured questionnaire to collect information on demographics, medical history, and lifestyle habits including smoking history. Questions about lifestyle habits focused on the time period ending one year prior to diagnosis for cases and on the year prior to the interview date for controls.

### 2.2. Genotyping Methods

DNA was extracted from the peripheral blood lymphocytes of each participating subject.* GSTM1* and* GSTT1* null alleles were identified using a multiplex-polymerase chain reaction- (PCR-) based method [15]. The polymorphic site at nucleotide 638 in exon 7 (Arg213His (^*^ *2 *allele), rs9282861) of the* SULT1A1* gene was genotyped by PCR-restriction fragment length polymorphisms (RFLP) analysis as described by Coughtrie et al. [16].* CYP1A1 *3′-flanking region* Msp*I polymorphism (*CYP1A1* ^*^ *2A* allele, rs4646903),* CYP2E1 Pst*I/*Rsa*I polymorphism (*CYP2E1* ^*^ *5B* allele, rs3813867 (*Pst*I)), and* CYP2E1 Dra*I (^*^ *5A* or ^*^ *6* alleles, rs6413432) were also determined by PCR-RFLP analyses. Quality control for each genotyping was performed in each experiment, and 10% of the total samples were randomly selected and reanalyzed with 100% concordance. All laboratory procedures were carried out blindly to case-control status.

### 2.3. Statistical Analysis

Hardy-Weinberg equilibrium (HWE) was tested for the control SNPs. Odds ratios (OR) of HCC and corresponding 95% confidence intervals (CI) according to analyzed polymorphisms were derived from unconditional multiple logistic regression models [17] using dominant model for carriers of the mutated allele, including terms for age and sex. When cell sizes were small (<5), exact logistic regression was used [18].

We also examined the possible confounding effect of smoking, alcohol, and chronic infection with HBV and/or HCV. However, models including these covariates yielded very similar results; thus, given the small numbers in some strata, only the age- and sex-adjusted estimates were presented.

Gene-gene interaction analysis was conducted, using as a reference group the homozygous wild-type individuals for both genes, while for gene-environment interaction analyses, the reference group was wild-type homozygotes not exposed to the environmental risk factor. Biological interaction between two genes was estimated using departure from additivity of effects as the criterion of interaction, as proposed by Rothman [19]. To quantify the amount of interaction, the attributable proportion (AP) due to interaction was calculated together with its 95% CI as described by Andersson et al. [20]. The AP due to interaction is the proportion of individuals among those exposed to the two interacting factors that is attributable to the interaction per se and it is equal to 0 in the absence of a biological interaction [19]. In order to test for more than multiplicative effect among two genes, the likelihood ratio test was used.

The paper has been written according to the STREGA guidelines [21].

## 3. Results

The demographics, clinical features and lifestyle habits of 221 HCC cases and 290 controls are reported in Table 1. Ever smokers were more common among cases, as well as infection with HBV and/or HCV (Table 1). The genotype frequencies were in HWE (*P* > 0.05).

Table 2 reports the distribution of the polymorphisms considered among HCC patients and controls. The carriers of* c2 *variant allele of* CYP2E1* ^*^ *5B *polymorphisms were less common in cases (1.8%) than in controls (6.9%) corresponding to an OR of 0.23 (95% CI: 0.06–0.71). Similarly, the frequency of the variant allele of* CYP2E1* ^*^ *6 *polymorphism was also less common among cases (1.0%) than controls (10%), with an OR of 0.08 (95% CI: 0.01–0.33) (Table 2). The selected polymorphisms of* CYP1A1*,* GSTM1*,* GSTT1*, and* SULT1A1* did not significantly influence susceptibility to HCC.

The gene-gene and gene-smoking interaction results are reported in Tables 3 and 4. We observed a borderline increased risk for HCC among carriers of combined* CYP1A1* ^*^ *2A* and* SULT1A1 *variant alleles as compared to the double wild-type homozygotes (OR = 1.67; 95% CI: 0.97–3.24) (Table 3). A significant interaction was reported between* GSTT1* and smoking (AP = 0.48; 95% CI: 0.001–0.815), with an OR of 3.13 (95% CI: 1.69–5.82) for* GSTT1* null genotype carriers who were smokers (Table 4). A borderline significant interaction was also observed for* SULT1A1* and smoking (AP = 0.36; 95% CI: −0.021–0.747), with an OR of 3.05 (95 CI: 1.73–5.40) for those* SULT1A1* variant allele carriers who were smokers (Table 4).

## 4. Discussion

Our study identified* CYP2E1* ^*^ *5B* and* CYP2E1* ^*^ *6 *variant alleles associated with a reduced risk of HCC. There was a borderline increased risk for HCC among carriers of combined* SULT1A1* and* CYP1A1* ^*^ *2A* variant alleles. The polymorphisms in* GSTT1* and* SULT1A1 *are associated with increased susceptibility to smoking-related HCC.

*CYP2E1* can activate N-nitrosamines and benzene contained in cigarette smoke [22] and is involved in alcohol-mediated generation of oxidative stress [23]. The expression of* CYP2E1* correlates with the generation of hydroxyethyl radicals and lipid peroxidation products [23]. The variant* CYP2E1* ^*^ *5B* and* CYP2E1* ^*^ *6* alleles are associated with increased transcription of* CYP2E1 *[24] that leads to development of HCC by promoting carcinogenesis. No significant association between* CYP2E1* ^*^ *6* and HCC was reported so far [25, 26], while contradictory results were reported for the* CYP2E1* ^*^ *5B* variant allele [27–35]. A recent meta-analysis did not find* CYP2E1* ^*^ *5B* c2 allele to be associated with HCC [36], also after stratifying among Asians and white. Studies conducted among white individuals, however, were few [26, 30, 35] and included limited numbers of cases.

The favorable effect of both* CYP2E1* polymorphisms on HCC development is not consistent with the biological premises implying a promoting role of a high activity enzyme. However, the only study previously conducted in an Italian population [35] on* CYP2E1* ^*^ *5B* c2 allele and HCC did report a similar association, indicating a favorable role of the variant allele against HCC which deserves further investigation.

Our results suggest that up to 48% and 36% of HCC cases among smokers carrying, respectively, variant* GSTT1 *and* SULT1A1* alleles occurred because of gene-smoking interaction. However, we could not further stratify these two results according to quantity of smoking, as numbers were low. Smoking is recognized as a risk factor for HCC [3–5] and, together with HBV and HCV, one of the major risk factors in Europe [37], and enzymes coded by* GSTT1* and* SULT1A1* have their role in the metabolism of tobacco carcinogens. There is therefore a biological ground for possible synergic carcinogenic effect.

There was a borderline synergic effect of* SULT1A1* and* CYP1A1* ^*^ *2A* polymorphisms in HCC carcinogenesis. The effect of* SULT1A1* polymorphism on HCC development has not been investigated so far. It has been reported, however, that an enzyme coded by* SULT1A1* variant allele has twofold lower catalytic activity in detoxifying the procarcinogens [38]. The biological significance of variant* CYP1A1* ^*^ *2A *variant allele is uncertain, but* CYP1A1* ^*^ *2A *has been reported to increase susceptibility to several cancer types, including lung, breast, and cervical cancer [39–41]. There is therefore a rationale for a synergic effect of these two SNPs in HCC carcinogenesis.

In our study, there was no significant association between HCC and alcohol. As most of the cases have hepatitis, this could lead them to stop drinking. Consequently we were unable to perform gene-alcohol interaction analysis. Secondly, we cannot exclude a selection bias. However, since the observed frequencies of the variant alleles of* CYP1A1* ^*^ *2A*, as well as the null genotypes of* GSTM1* and* GSTT1*, were in line with those previously reported in the Italian population, a major impact of such bias is unlikely [35, 42]. Thirdly, our study was underpowered to perform gene-gene and gene-interaction analysis.

In conclusion, we report that* CYP2E1* ^*^ *5B* and* CYP2E1* ^*^ *6 *polymorphisms have a favorable effect on the development of HCC, while the polymorphisms in* GSTT1 *and* SULT1A1 *may increase HCC susceptibility among smokers.

## Figures and Tables

**Table 1:** Distribution of 221 cases of hepatocellular carcinoma (HCC) and 290 controls according to selected factors.

	HCC cases (*N* = 221)		Controls (*N* = 290)	
	*N*	(%)	*N*	(%)
Age (years)				
<60	43	(19.5)	105	(36.2)
60–69	80	(36.2)	78	(26.9)
≥70	98	(44.3)	107	(36.9)
Sex				
Male	160	(72.4)	176	(60.7)
Female	61	(27.6)	114	(39.3)
Smoking^a^				
Never	85	(39.2)	173	(59.7)
Ever	133	(60.8)	117	(40.3)
*P* ^b^ < 0.001				
Hepatitis^a,c^				
No	64	(29.0)	282	(97.9)
Yes	157	(71.0)	6	(2.1)
*P* ^b^ < 0.001				

^a^The sum does not add up to the total because of some missing values.

^b^ *P* values from *χ* ^2^ test.

^c^Hepatitis was defined as history of hepatitis B and/or C.

**Table 2:** Distribution of cases and controls, odds ratios^a^ (OR), and 95% confidence intervals (CI) for hepatocellular carcinoma (HCC) according to selected polymorphisms.

	HCC cases (*N* = 221)		Controls (*N* = 290)		OR (95% CI)
	*N*	(%)	*N*	(%)	
*CYP1A1* ^*^ *2A *					
wt/wt	165	(74.7)	226	(77.9)	1^b^
wt/mt and mt/mt	56	(25.3)	64	(22.7)	1.21 (0.80–1.84)
*CYP2E1* ^*^ *5B*					
*c*1/*c*1	217	(98.2)	270	(93.1)	1^b^
*c*1/*c*2 and *c*2/*c*2	4	(1.8)	20	(6.9)	0.23 (0.06–0.71)
*CYP2E1* ^*^ *6* ^c^					
wt/wt	204	(99.0)	261	(90.0)	1^d^
wt/mt and mt/mt	2	(1.0)	29	(10.0)	0.08 (0.01–0.33)
*GSTM1* ^c^					
Present	96	(47.8)	139	(48.1)	1^b^
Null	105	(52.2)	150	(51.9)	0.99 (0.68–1.43)
*GSTT1* ^c^					
Present	141	(70.1)	220	(76.1)	1^b^
Null	60	(29.9)	69	(23.9)	1.35 (0.89–2.05)
*SULT1A1 *					
wt/wt	132	(59.7)	180	(62.1)	1^b^
wt/mt and mt/mt	89	(40.3)	110	(37.9)	1.22 (0.84–1.77)

^a^Adjusted for age and sex.

^b^Reference category.

^c^The sum does not add up to the total because of some missing values.

^d^Calculated from exact logistic regression analysis.

wt: wild-type allele.

mt: variant-type allele.

**Table 3:** Effect of the genes-gene interaction on the development of hepatocellular carcinoma.

	Cases : controls	OR^a^ (95% CI)	*P* ^b^	AP (95% CI)
*GSTM1* × *CYP1A1* ^*^ *2A *				
Present/wt homozygote	74 : 113	1^c^		
Null/wt homozygote	78 : 112	1.05 (0.69–1.61)		
Present/any mt	22 : 26	1.32 (0.69–2.53)		
Null/any mt	27 : 38	1.04 (0.58–1.88)	0.521	nc
*GSTM1* × *GSTT1 *				
Present/present	66 : 103	1^c^		
Null/present	75 : 117	0.97 (0.63–1.50)		
Present/null	30 : 36	1.28 (0.71–2.30)		
Null/null	30 : 33	1.39 (0.77–2.53)	0.773	0.109 (−0.603; 0.820)
*GSTM1* × *SULT1A1 *				
Present/present	53 : 84	1^c^		
Null/present	67 : 96	1.09 (0.68–1.76)		
Present/null	43 : 55	1.40 (0.81–2.41)		
Null/null	38 : 54	1.22 (0.70–2.13)	0.566	nc
*GSTT1* × *CYP1A1* ^*^ *2A *				
Present/wt homozygote	108 : 169	1^c^		
Null/wt homozygote	44 : 56	1.20 (0.74–1.93)		
Present/any mt	33 : 51	0.98 (0.58–1.63)		
Null/any mt	16 : 13	2.00 (0.91–4.41)	0.289	0.414 (−0.149; 0.976)
*GSTT1* × *SULT1A1 *				
Present/wt/wt	81 : 138	1^c^		
Null/wt/wt	39 : 42	1.59 (0.94–2.70)		
Present/any mt	60 : 82	1.40 (0.90–2.20)		
Null/any mt	21 : 27	1.48 (0.77–2.84)	0.347	nc
*SULT1A1* × *CYP1A1* ^*^ *2A *				
wt homozygote/wt homozygote	99 : 138	1^c^		
mt carrier/wt homozygote	66 : 88	1.14 (0.75–1.74)		
wt homozygote/mt carrier	33 : 62	1.09 (0.64–1.85)		
mt carrier/mt carrier	23 : 22	1.67 (0.97–3.24)	0.493	0.269 (−0.330; 0.867)

OR: odds ratio; CI: confidence interval; AP: attributable proportion; nc: not calculable.

^a^Adjusted for age and sex.

^b^ *P* from test for multiplicative interaction.

^c^Reference category.

wt: wild-type allele.

mt: variant-type allele.

**Table 4:** Effect of the gene-smoking interaction on the development of hepatocellular carcinoma.

	Cases : controls	OR^a^ (95% CI)	*P* ^b^	AP (95% CI)
*CYP1A1* ^*^ *2A* × smoking^c^				
wt homozygote/no	65 : 136	1^d^		
wt homozygote/yes	98 : 90	2.16 (1.29–3.36)		
mt carrier/no	20 : 37	1.08 (0.58–2.03)		
mt carrier/yes	35 : 27	2.81 (1.51–5.23)	0.680	0.201 (−0.328; 0.730)
*GSTM1* × smoking^c^				
Present/no	31 : 91	1^d^		
Present/yes	62 : 48	4.01 (2.20–7.28)		
Null/no	48 : 81	1.82 (1.05–3.18)		
Null/yes	57 : 69	2.34 (1.32–4.23)	0.004	nc
*GSTT1* × smoking^c^				
Present/no	59 : 129	1^d^		
Present/yes	81 : 91	1.86 (1.17–2.97)		
Null/no	20 : 43	0.99 (0.53–1.86)		
Null/yes	38 : 26	3.13 (1.69–5.82)	0.230	0.480 (0.001; 0.815)
*SULT1A1* × smoking^c^				
wt homozygote/no	52 : 103	1^d^		
wt homozygote/yes	78 : 77	1.93 (1.19–3.14)		
mt carrier/no	33 : 70	1.01 (0.59–1.75)		
mt carrier/yes	55 : 40	3.05 (1.73–5.40)	0.250	0.363 (−0.021; 0.747)

OR: odds ratio; CI: confidence interval; AP: attributable proportion; nc: not calculable.

^a^Adjusted for age and sex.

^b^ *P* from test for multiplicative interaction.

^c^The sum does not add up to the total because of some missing values.

^d^Reference category.

wt: wild-type allele.

mt: variant-type allele.
